# Preclinical Assessment Addressing Intravenous Administration of a [^68^Ga]Ga-PSMA-617 Microemulsion: Acute In Vivo Toxicity, Tolerability, PET Imaging, and Biodistribution

**DOI:** 10.3390/molecules26092650

**Published:** 2021-04-30

**Authors:** Vusani Mandiwana, Lonji Kalombo, Rose Hayeshi, Jan Rijn Zeevaart, Thomas Ebenhan

**Affiliations:** 1 Centre for Nanostructures and Advanced Materials CeNAM, Chemical Cluster, Advanced Functional Materials, Council for Scientific and Industrial Research, Pretoria 0001, South Africa; lkalombo@csir.co.za; 2 Department of Science and Technology, Preclinical Drug Development Platform, North West University, Potchefstroom 2520, South Africa; Rose.Hayeshi@nwu.ac.za (R.H.); janrijn.zeevaart@necsa.co.za (J.R.Z.); thomas.ebenhan@gmail.com (T.E.); 3 Radiochemistry, South African Nuclear Energy Corporation (Necsa), Pelindaba, Brits 0240, South Africa; 4 Department of Nuclear Medicine, University of Pretoria and Steve Biko Academic Hospital, Pretoria 0001, South Africa; 5 Preclinical Imaging Facility, NuMeRI, Pelindaba, Brits 0242, South Africa

**Keywords:** biodistribution, ^68^Ga, [^68^Ga]Ga-PSMA-617-ME, in vivo, microemulsion, microPET/CT, prostate cancer, PSMA-617, toxicity

## Abstract

It has been herein presented that a microemulsion, known to be an effective and safe drug delivery system following intravenous administration, can be loaded with traces of [^68^Ga]Ga-PSMA-617 without losing its properties or causing toxicity. Following tolerated IV injections the capability of the microemulsion in altering [^68^Ga]Ga-PSMA-617 distribution was presented at 120 min post injection based on its ex vivo biodistribution results.

## 1. Introduction

A microemulsion (ME) is a translucent, optically isotropic colloidal system made up of various components including water, oil, and an amphiphile forming spontaneously with an average droplet diameter of 10 to 140 nm [1]. It is generally understood and established that the pharmacological and medicinal application of microemulsions is connected to certain advantages: I) They are thermodynamically stable and therefore need minimal energy to formulate; II) they facilitate a more controlled drug release and targeting; III) they improve the efficacy of the drug, therefore allowing the total dose to be reduced; and IV) they are essential tools in reducing drug side effects [2,3,4]. Such drug-entrapped or activated microemulsions are used as drug delivery systems which can be administered *inter alia*, topically, orally, parenterally, and through nasal routes [5]. The preparation of an activated ME typically involves dissolving an appropriate amount of a drug in either an oil or aqueous phase containing surfactant and mixing the solubilized mixture with co-surfactant to form the ME at specific temperatures [6]. Proposals for an intravenously (IV) administered ME-based system suggest a composition of particular lipids/oils, medium-chain triglycerides, polyethylene glycol (PEG), and water [6,7,8]. The small droplet size of the ME enables the delivery system to escape uptake and phagocytosis by the reticuloendothelial system and will increase the circulation time of the entrapped drug. Furthermore, activated ME systems are well capable of a slower release of drugs from their core averting a burst release observed with conventional formulations [2].

The rationale behind encapsulation of radiopharmaceuticals into a ME delivery system is that it offers potential prospects in the approach of diagnosing and/or treating patients with slow-release radiotherapeutics, therefore tailoring their overall uptake and residence time and potentially reducing radiation burden to vulnerable organs such as the kidneys or glandular organs. It is also plausible that the use of MEs would aid in better delivery mechanisms through addition of PEG thereby increasing systemic circulation time. Testing the ME for its acute toxicity is a necessary prerequisite investigation to meet the safety aspects for animals. The benefits of this assessment are: (I) Awareness of clinical signs attributable to injectable doses of the test substance (i.e., large dose volume, too high activity concentration of the radioactive agent mass); (II) time of onset and remission of those signs; and (III) possible determination of a minimum lethal dose or of effects leading to death or recovery [9]. We have been following the concept of developing a ME-based delivery system activated with a small-sized radiolabeled inhibitor targeting prostate-specific membrane antigen called vipivotide tetraxetan (also known as PSMA-617). Structurally, it comprises of the targeting moiety—a urea-containing tripeptide with high affinity to PSMA, and the chelator tetraxetan (DOTA). Radiolabeled PSMA-617 analogues are currently successfully utilized as theranostic precursor molecules for imaging and/or therapy of prostate cancer [10]. Tetraxetan is hereby of high value to warrant the formation of thermodynamically and kinetically stable complexes when radiolabeled with radionuclides such as gallium-68 (^68^Ga), iodine-131 (^131^I), lutetium-177 (^177^Lu), and yttrium-90 (^90^Y) [10]. The application of ^177^Lu-based targeted radioligand therapy (TRT) is currently the most reported form; i.e., [^68^Ga]Ga-PSMA would be a considerable option to diagnose prostate cancer followed by the treatment therapy option of [^177^Lu]Lu-PSMA-617-TRT. Success with TRT is based on the fact that PSMA expression increases in metastatic and hormone refractory prostate cancer, which makes it an ideal target for positron emission tomography/computed tomography (PET/CT) imaging and therapy [11].

This being an exemplifying strategy, a ME-based delivery system was hypothesized to aid in rendering [^68^Ga]Ga-PSMA-617 more efficient in vivo, once it becomes entrapped within a ME, due to reduced side effects and toxicity to the kidneys and other non-target organs. This delivery system should ideally distribute the incorporated [^68^Ga]Ga-PSMA-617 to the prostate cancer through passive targeting via the enhanced permeability and retention (EPR) effect therefore reducing toxic uptake or accumulation in the kidneys. In a preliminary study a microemulsion system entrapped with [^68^Ga]Ga-PSMA-617 was accomplished as demonstrated by the characterization of the activated ME (i.e., [^68^Ga]Ga-PSMA-617-ME). Such a loaded ME showed negligible in vitro toxicology. A first attempt of intravenous administration in mice was achieved but it was concluded that acute toxicity and tolerability should be investigated prior to further in vivo applications such as molecular imaging. Obtaining more information on the toxicity profile of the ME formulation in conjunction with clinically approved [^68^Ga]Ga-PSMA-617 could foster understanding the potential of MEs for clinical use within nuclear medicine. Hence, we herein present an optimized radiolabeling approach that yielded comparable radiolabeled and characterized products (i.e., [^68^Ga]Ga-PSMA-617 vs. [^68^Ga]Ga-PSMA-617-ME) for an original in vivo assessment. The acute toxicity following intravenous compound injections in BALB/c mice was determined also considering gross necropsy, clinical biochemistry, and histopathologic observations. Dynamic [^68^Ga]Ga-PSMA-617-ME-microPET/CT imaging and *post mortem* organ biodistribution studies were performed to learn of any hypothetically altered pharmacokinetics or organ uptake concerning [^68^Ga]Ga-PSMA-617-ME and whether a prolonged systemic residence time can be demonstrated.

## 2. Results

### 2.1. Preparation and Radioanalysis of [^68^Ga]Ga-PSMA-617 and [^68^Ga]Ga-PSMA-617-ME

For the investigation of acute in vivo toxicity a total of 138–357 MBq (concentration 68–179 MBq/mL) Ga-68 activity was yielded by eluate fractionation. In a similar fashion, for PET imaging purposes, the starting activity of 588–757 MBq was eluted, buffered with 2.5 M sodium acetate (pH 4.0), and used straightforward for radiolabeling with 20 µg PSMA-617. Further optimization of the purification process post radiolabeling provided robust [^68^Ga]Ga-PSMA-617 yields (LE > 95%, RCP: >98%; *n* = 5). The radio-HPLC based product identification (retention time 6.27 ± 0.51 min) was more consistent to previously published results [11]. Low evidence of colloidal-^68^Ga was observed (>5.0%, ITLC-based). Expected amounts of unretainable radioactivity occurred on glass surfaces, C18-SepPak- and sterile filter material (8.5–14.5%). The [^68^Ga]Ga-PSMA-617 preparations achieved acceptable product sterility and quantities (RCY (uncorrected for decay): 325 to 420 MBq (15–19 GBq/µmol), total volume: 0.5–0.75 mL saline; 25 % ethanol; *n* = 5). This product solution was further formulated in 10 mL PBS, pH 6.8 to dilute the ethanol content < 2% for administration. Respectively produced [^68^Ga]Ga-PSMA-617 radioactivity formed part of a 10 mL microemulsion formulation (8.28% (*v/v*); i.e., [^68^Ga]Ga-PSMA-617-ME or ^68^Zn-PSMA-617 (i.e., decayed [^68^Ga]Ga-PSMA-617 samples); other composites (*v/v*) as follows: (1) 0.17% lauric acid-PEG 4000, (2) 8.28% ethanol, (3) 41.39% PVA, (4) 41.39% sodium oleate, (5) 0.17% d-α-tocopherol, and (6) 0.33%Tween 80. The ME (blank) consisted of the same percentage of composites from 1–6 and 8.28% saline, accordingly. For imaging the activity concentrations (MBq/mL) used for [^68^Ga]Ga-PSMA-617 and [^68^Ga]Ga-PSMA-617-ME were 23.5 ± 8.6 and 17.5 ± 6.9, respectively.

### 2.2. Characterization of ^68^Zn-PSMA-617 and ^68^Zn-PSMA-617-ME

First, measurement of ME (blank) and ^68^Zn-PSMA-617-ME samples for droplet size, distribution (PDI) and surface charge (Zeta potential) demonstrated the following differences: (I) The average particle size of the microemulsion (72.23 ± 0.18 nm) was significantly higher compared to ^68^Zn-PSMA-617-ME (27.61 ± 1.11 nm; *p* < 0.01), which implied that the addition of saline increased the droplet particle size whereas the addition of ^68^Zn-PSMA-617-ME maintained a narrow droplet size (average particle size ME lacking saline ranged between 20 and 40 nm with a distribution between 0.10 and 0.30 [11]); (II) the PDI for the ^68^Zn-PSMA-617-ME (0.53 ± 0.01) was higher than that of the ME (0.26 ± 0.01, *p* < 0.001) which may have been a result of the addition of ^68^Zn-PSMA-617 leading to the formation of a system with multiple particle sizes; and (III) neutral conditions (pH ~7.0) caused no differences in the negative zeta potential between ME and ^68^Zn-PSMA-617-ME (both <−5.0 mV). Second, Table 1 summarizes the results comparing the characterization of ^68^Zn-PSMA-617 samples with ^68^Zn-PSMA-617-ME formulations *(n* = 3). Different parameters including the masses, volumes, concentrations, and types of excipients were optimized to obtain physiological solutions with average particle size ranging between 22.00 and 59.00 nm, with PDI values ≤ 0.25 and a fitting negative zeta potential (<−19 mV), thus meeting the criteria for an intravenous administration.

### 2.3. Acute In Vivo Toxicity and Tolerability of Intravenous Administration of ^68^Zn-PSMA-617-ME

#### 2.3.1. Food Consumption and General Appearance

During the 14-day study duration, none of the BALB/c mice which were intravenously injected with either the ME (control) or ^68^Zn-PSMA-617-ME died or exhibited any adverse events or abnormalities. There were no unexpected changes in the food consumption; the intake was constant throughout the study at a rate of 3.74 g ± 0.32 per mouse per day. There were also no abnormal changes observed in the coat condition, respiration, mobility, and behavior of any kind, during the study.

#### 2.3.2. Body Weight and Organ Necropsy and Histology

The microemulsion treatment group was assigned mice with a starting mean weight of 18.05 g ± 2.55; the lowest and highest starting weights being 15.93 g and 22.90 g respectively. The ^68^Zn-PSMA-617-ME treatment group had a starting mean weight of 19.86 g ± 1.21, with the lowest and highest weights being 18.53 g and 21.84 g. Within 48 h post injection 4/5 (80%) mice that received the ME and 2/5 (40%) mice injected with ^68^Zn-PSMA-617-ME experienced noticeable individual weight loss (*P* < 0.05) with an average weight loss of 2.31% ± 0.32 and 1.37% ± 0.43, respectively. In addition, the initial (day 0) and final body weight (day 14) were compared in mice between the two test groups; from 48 h onwards 9/10 (90%) animals gradually gained weight, indicating acceptable tolerability of the injection. Only 1/10 mice (10%) which received a ME completed the study with a weight loss of 1.36% within 14 days. The organ weights were determined after euthanasia following animal dissection and gross organ necropsy. The results comparing the two study groups are summarized in Table 2. Following veterinary examination, all the listed organs were of the expected size, weight, and color, which were of general healthy gross appearance. The histopathology assessment returned negative for any treatment-related toxicity and showed no abnormalities or malfunction of the organs. The microscopic evaluation of selected organs showed no diagnostically significant changes in any of the provided heart, lungs, liver, kidneys, spleen, brain, stomach, small intestines, large intestines, and pancreas sections which were evaluated.

#### 2.3.3. Clinical Biochemistry

During animal dissection, 14 days post injection, available blood was sampled and further subjected to biochemical profiling. The measurements to test for technical standards were obtained from published literature [12,13]. The control measurement was used in cases where the technical standard was not reported. The test results are summarized in Table 3.

^68^Zn-PSMA-617-ME intravenous administration showed elevated levels of bilirubin and ALT (upper normal limit) as well as decreased levels of Na^+^ (lower normal limit), lowered serum lipids—especially for cholesterol and triglycerides. When the ME was administered, blood samples presented with normal levels except for individual findings with elevated albumin (upper normal limit), high phosphatase levels lowered amylase (lower normal limit), and low creatinine and triglycerides.

### 2.4. MicroPET/CT Imaging

Real time in vivo biodistribution was investigated after injecting [^68^Ga]Ga-PSMA-617 and [^68^Ga]Ga-PSMA-617-ME into BALB/c mice. The injected activity across all the mice was in the range of 0.37–5.60 MBq (0.27–0.36 nmol/ mouse). A decayed sample of the injected [^68^Ga]Ga-PSMA-617-ME solution was characterized as follows: particle size range; 30.48 to 58.43 nm and zeta potential range; −19.25 to −27.96 mV. Similarly, [^68^Ga]Ga-PSMA-617 had a droplet size and zeta potential which ranged from 22.25 to 25.79 nm and −22.10 to −27.22 mV, respectively.

**Figure 1:**
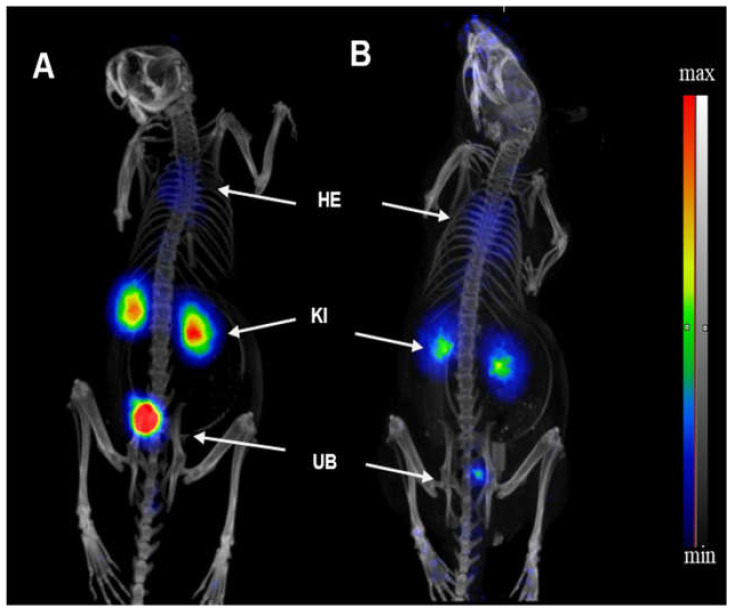
Representative maximum intensity projection microPET/CT images: Healthy BALB/c mice received a 0.1 mL intravenous injection of [^68^Ga]Ga-PSMA-617-ME via the lateral tail vein. PET/CT image acquisition data from (**A**) up to 40 min post injection was reconstructed and displayed and compared to (**B**) [^68^Ga]Ga-PSMA-617-ME PET/CT at 90–120 min. Recognized ^68^Ga-activity is visible in the myocardium (HE), both kidneys (KI), and the urinary bladder (UB).

Maximum intensity projection microPET/CT images representing [^68^Ga]Ga-PSMA-617-ME activity distribution up to 40 min compared to 90–120 min post injection are depicted in Figure 1. Qualitative image analysis demonstrated significant radioactivity in the kidneys soon after injection, notable presence of radioactivity in the myocardium is evident in both images. Similar to [^68^Ga]Ga-PSMA-617 (not shown), the renal clearance route was confirmed by visible early onset bladder activity for [^68^Ga]Ga-PSMA-617-ME. Similar distribution profiles have been observed in mice injected with ^68^Ga-DOTA labelled peptides as reported by Umbricht et al., 2017. However, both, [^68^Ga]Ga-PSMA-617-ME and [^68^Ga]Ga-PSMA-617, showed an attributed clearance profile for small-sized polar radiopharmaceuticals, with predominant renal clearance [14].

**Figure 2:**
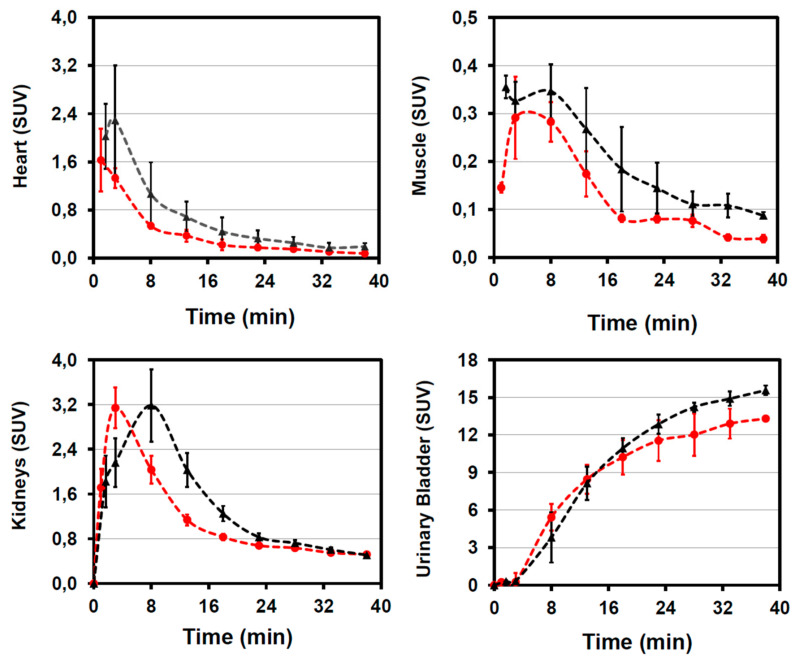
SUV derived analysis of time-activity curves for heart, muscle, kidney, and bladder displayed up to 38 min after intravenous injection of [^68^Ga]Ga-PSMA-617 (black) and [^68^Ga]Ga-PSMA-617-ME (red). Results are expressed as mean (±standard error of mean (SEM); *n* = 4).

Following dynamic [^68^Ga]Ga-PSMA-617- and [^68^Ga]Ga-PSMA-617-ME-microPET/CT image reconstruction image quantification included drawing of time-activity-curves (TAC) (SUV, i.e., tissue concentration over time) comparing both compounds over 38 min as displayed in Figure 2. Heart TACs were used for calculation of initial tissue concentration (SUV_t0_), pharmacological half-lives (pT½), and blood pool clearance rates (∆t-bp): values for [^68^Ga]Ga-PSMA-617-ME (SUV_t0_ = 1.44 ± 0.27, pT½ = 8.3 ± 0.3 min, ∆t-bp = −0.084 ± 0.003 kBq/cm^3^ × min^−1^) were similar to [^68^Ga]Ga-PSMA-617 (SUV_t0_ = 2.20 ± 0.24, pT½ = 8.6 ± 1.3 min, ∆t-bp = −0.082 ± 0.013 kBq/cm^3^ × min^-1^). Muscle TAC indicated similar, favorably low presence of both tracers (SUV < 0.4) early on after injection, further decreasing to SUV values of 0.05–0.1. TACs derived from kidneys demonstrated similar curves however the peak radioactivity for [^68^Ga]Ga-PSMA-617-ME (SUV 3.1 ± 0.3) was observed as early as 3 min post injection and decreased exponentially to five-fold less concentrations (0.63 ± 0.03) over the following 20 min. The radioactivity was subsequently eliminated as represented in increasing the bladder activity culminating to SUV ranging 11.6–13.3 at 23–38 min. In comparison, [^68^Ga]Ga-PSMA-617 kidney activity peaked at 8 min post injection (SUV 3.2 ± 0.4) and decreased exponentially to 4.5-fold less concentrations (0.72 ± 0.07) over the following 20 min. Consequently, kidney activities were significantly higher for [^68^Ga]Ga-PSMA-617 vs. [^68^Ga]Ga-PSMA-617-ME at 8–28 min (*P* < 0.05). In addition, the bladder accumulation for [^68^Ga]Ga-PSMA-617 followed a similar TAC pattern to that of [^68^Ga]Ga-PSMA-617-ME. However, it is observed that the SUVs were peaking significantly higher at 14.2–15.8 at 28–38 min (*P* = 0.004).

### 2.5. Ex Vivo Biodistribution

**Figure 3:**
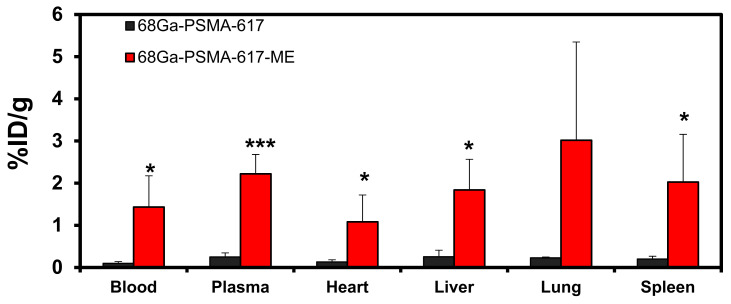
Ex vivo biodistribution (%ID/g) of [^68^Ga]Ga-PSMA-617 (black) and [^68^Ga]Ga-PSMA-617-ME (red) in blood and prefunded organs at 120 min after intravenous injections. Radioactive organ samples and radioactivity standards were measured using high-performance gamma counting. Results are expressed as mean (±SEM; *n* = 4). * *P* < 0.05; *** *P* < 0.001.

The ex vivo biodistribution analysis was performed to determine the differences of the radioactivity pattern of [^68^Ga]Ga-PSMA-617-ME compared to [^68^Ga]Ga-PSMA-617. The [^68^Ga]Ga-PSMA-617-ME showed much higher presence in whole blood (and plasma), calculating up to 15-fold differences (*P* < 0.001) compared to the presence of [^68^Ga]Ga-PSMA-617 at 120 min post injection (Figure 3). It is therefore possible that when [^68^Ga]Ga-PSMA-617 is entrapped into polymeric nanoparticles (i.e., microemulsion, micelles etc.), it may exhibit delayed blood clearance. The significantly higher levels of [^68^Ga]Ga-PSMA-617-ME is also reflected in all blood-rich organs (heart, liver, and spleen; *P* < 0.05) as well as the trend in lung tissue (*P* = 0.099).

**Figure 4:**
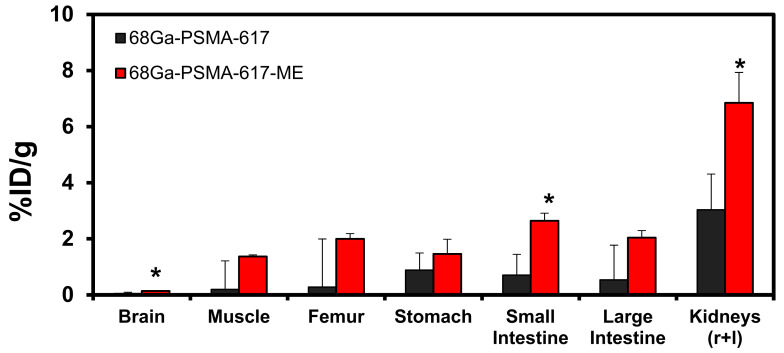
Organ and tissue biodistribution (%ID/g) of [^68^Ga]Ga-PSMA-617 (black) and [^68^Ga]Ga-PSMA-617-ME (red) 120 min after intravenous injection. Radioactive organ samples and radioactivity standards were measured using high-performance gamma counting. Results are expressed as mean (±SEM; *n* = 4). * *P* < 0.05.

As displayed in Figure 4, significantly higher amounts of [^68^Ga]Ga-PSMA-617-ME than [^68^Ga]Ga-PSMA-617 were also observed for the kidneys and small intestines (*P* < 0.05). The cumulative kidney uptake of [^68^Ga]Ga-PSMA-617-ME was two-fold higher than that recorded on [^68^Ga]Ga-PSMA-617 (averaged 6.85%ID/g vs. 3.20%ID/g respectively). Comparison of the measurements for the other organs were not statistically significant (*P* > 0.05). The biodistribution pattern for [^68^Ga]Ga-PSMA-617 represented in the kidneys (3.20%ID/g) and stomach (1.08%ID/g) was similarly reported by Benesova et al., 2015 [15]. Other studies also reported that the highest [^68^Ga]Ga-PSMA-617 uptake was confirmed in kidneys, liver, spleen, and stomach [14,16].

## 3. Discussion

Although ME are quite effective in delivering drugs via various routes of administration, very sparse information is reported on the readiness for safe intravenous administration of ME-entrapped drugs or radiopharmaceuticals, in particular. Currently, urea-containing peptides-based radiopharmaceuticals such as PSMA-617 are emerging as tools for theranostic nuclear medicine procedures such as alpha-particle therapy and TRT. To this end, being polar, small molecules, the pharmacokinetic profile and some off-target pharmacology may be enhanced by using a surfactant-based delivery system i.e., microemulsions. For both, nuclear imaging and possible radioendotherapy applications, tetraxetan is the most studied and acceptable chelator for the complexation of radiometals (i.e., ^68^Ga, ^177^Lu, and ^90^Y). Opting for PSMA-617, a highly potent PSMA inhibitor, was three-fold [17]: (I) endogenous PSMA-617 was tested in a repeated dose toxicology study in male rats for 22 days. The test item was administered once weekly by IV bolus injection on days 1, 8, 15, and 22. No signs of local or systemic intolerance reactions were observed at 400 mg/kg. Thus, any plausible toxicity would have to be attributed to the changes coming from its ME entrapment; (II) unlike other such molecules it showed excellent extended radiolytic stability, and (III) if successful, a compound with relevance to targeted radionuclide therapy fits the future concept to address the suitability of ME-based formulation. The use of [^68^Ga]Ga-PSMA-617 in this study should be noted as a preliminary, yet not exhaustive example of a radiolabeled compound for the assessment towards potential benefits of ME. Conceptually, pharmacokinetic (PK) alterations to a known radiopharmaceutical like PSMA-617 may not be required to begin with, but could be considered advantageous to other biomolecules with more vulnerable properties. For both the radiosynthesis approach and ME-entrapment, it becomes imperative to meet the criteria for radiopharmaceutical preparation and formulation to subsequently perform initial preclinical assessments.

### 3.1. Characterization of ^68^Zn-PSMA-617 and ^68^Zn-PSMA-617-ME

High zeta potential is an indicator of the stability of the formulation and aids in predicting particle aggregation and/or phase precipitation [18]. Therefore these results may indicate that a low negative charge might not be a good indicator of microemulsion stability. Similar studies report stable microemulsion formulations at low negative charge zeta potentials [18]. Measurement of zeta potential also serves as a predictor of the storage stability of a colloidal system. Particle aggregation is less likely to occur in charged particles with high zeta potential due to electric repulsion [19]. This is true for kinetically stable emulsions (i.e., nanoemulsions), whereas in case of thermodynamically stable emulsions (i.e., microemulsions), only a measurable change in one of the variables (concentration, temperature or pressure) could lead to a change in the physical characteristics of the microemulsion. We observed no phase separation and the microemulsion formulations remained translucent even after storage for over 10 weeks at room temperature. The bigger the zeta potential of the suspension, the more likely it is to be stable because the charged particles repel each other and therefore overcome the natural tendency to aggregate and wherein it is accepted that zeta potentials more than ± 30.00 mV are sufficient for good electrostatic stabilization [19]. The ambient room temperature did not affect the physical stability of the formulations, more specifically, it did not influence phase separation or clarity during the study

### 3.2. Acute In Vivo Toxicity and Tolerability of Intravenous Administration of ^68^Zn-PSMA-617-ME

It is difficult to say whether the weight loss was influenced by the treatment compounds per se considering the weights of each mouse which was enrolled into the treatment groups. A linear regression model allowed us to determine whether the treatment, either ME (blank) or ^68^Zn-PSMA-617-ME, had any significant effect on the animals’ ability to sustain their body weight. The model indicated that the effect of ^68^Zn-PSMA-617-ME was statistically different (*P* < 0.0001) to the ME (blank) treatment group in the rate of growth and therefore it could be concluded that the two groups had statistically significant differences in body weight profiles over the 14-day study period, based on the information for *n* = 5 animals per treatment group and considering the variances in starting body weights. Even though an individual animal may not show a linear pattern of growth over time, the average growth of the groups seemed to be relatively linear, and therefore comparison of linear trends seemed to be appropriate. No significant differences in weight were found between the organs of the different treatment groups (*P* > 0.05; *n* = 5). This suggests no abnormal growth reaction or otherwise changes to organ appearance due to treatment with the ^68^Zn-PSMA-617-ME compared to ME (blank).

Elevation to constantly high alanine transaminase/aspartate transaminase ALT/AST levels may be associated with the destruction of hepatocytes and subsequent liver damage [13]. In literature, elevated AST levels in mice are also reported to be common [13]. An increase in both ALT and AST levels could also indicate liver injury or malfunction. Based on the gross necropsy observation and the histopathology report, this was not the case, as the liver samples did not exhibit any abnormalities or injury. Despite the difference between the treatment compounds, the results suggested no liver damage and the monitored study animals remained healthy throughout the experimental period. Creatinine levels were lowered in the test groups which could be an indication of clearance of creatinine by the kidneys and indication of good health in mice [12]. Statistical analysis (wherever the sample size allowed it) demonstrated insignificant differences for the comparison between groups; however, note that the results of the test should still be taken as indicative and preliminary since the number of usable animal samples was minimal. Having the lowest acceptable sample sizes could mean that patterns of differences can often not be detected and a larger sample size would be highly recommended. Using ME delivery systems that are safe and compatible with IV administration is a rather novel research field. Studies like this may provide clarity on whether the use of ME-radiopharmaceuticals have prospects (especially with no precedence investigation towards intravenous injections) or may lack benefit for future clinical settings. However, the preclinical setting addressing drug development aspects may be supported by allowing for a more efficient study design.

### 3.3. MicroPET/CT Imaging

Mice injected with [^68^Ga]Ga-PSMA-617-ME appeared to show slightly different retention of radioactivity in the kidneys as compared to those injected with [^68^Ga]Ga-PSMA-617, taking into account that lower radioactivity may have been injected and also the possibility that the microemulsion may delay biodistribution of [^68^Ga]Ga-PSMA-617. Small radiolabeled PSMA ligands are indeed excreted primarily via the urinary system and will collect in the bladder; small amounts are excreted via the hepatobiliary system [17]. The comparison of early and late images resulting from [^68^Ga]Ga-PSMA-617-ME distribution confirms such behavior, with no other organs being visualized that would emphasize [^68^Ga]Ga-PSMA-617-ME accumulation. Any potential accumulation of urea-based ^68^Ga-labeled PSMA ligands in the urinary system is partially due to the path of excretion of the agent and specific uptake from the expression of PSMA in mouse proximal renal tubules [20]. Thus, in this study, the high kidney uptake mainly represents renal excretion of [^68^Ga]Ga-PSMA-617(-ME)—a generally expected clearance behavior for small, polar peptide conjugates [15].

### 3.4. Ex Vivo Biodistribution

Prolonged circulation and protection of [^68^Ga]Ga-PSMA-617 by a microemulsion delivery system should ideally reduce residence time and therefore alter clearance of [^68^Ga]Ga-PSMA-617 favorably from the kidneys. Additionally, PSMA is also expressed physiologically in certain tissues, i.e., small intestines, colon, proximal renal tubules, kidneys, liver, spleen, and salivary glands [17]. This means that radiation dose is delivered to these organs when [^68^Ga]Ga-PSMA-617 is used for radionuclide targeting. This may affect the off target profile considerations and alter the properties for a safe dose that can be delivered without causing damage to the normal tissue [21]. However, for prostate cancer [^68^Ga]Ga-PSMA-617 would be expected to show a high tumor-to-background ratio compared to the surrounding tissue [17].

Noteworthy, given the injectable activity concentration, this study was limited to high quality imaging for up to 120 min post injection drawing a challenge to the biodistribution profile as an early on imaging time may not be sufficient to draw adequate conclusions. Toward future investigations the half-life of ^68^Ga may also be too challenging in microPET/CT imaging procedures where radioactivity decays quite rapidly and is paired with lower radioactivity for administration (diluted by way of creating the [^68^Ga]Ga-PSMA-617-ME). On a more strategic note, in particular for the preclinical setting of studying MEs in vivo other diagnostic radionuclides such as copper-64 may be advantageous as the proposed PK of certain biomolecules and may be better matched with the half-life of copper-64 (12.7 h) compared to that of gallium-68 (68 min).

## 4. Materials and Methods

### 4.1. Materials

Purchased reagents from Sigma Aldrich (St Louis, MO, USA) in the highest purity available: Tween 80, lauric acid, sodium oleate, d-α-tocopherol, polyvinyl alcohol (PVA) (97–98% hydrolyzed; molecular weight range 13–23kDa), and PEG-4000. All these excipients are approved by the Food and Drug Association and considered safe for both oral and intravenous administration [22]. Other chemicals, reagents, and solvents required for radiolabeling (i.e., ultrapure, metal-free water) and radioanalysis of the compounds were of analytical grade and were purchased from Sigma Aldrich (St Louis, MO, USA). High-performance liquid chromatography (HPLC)-grade ethanol was purchased from Radchem Products Inc. (Orlando Park, IL, USA). Vipivotide tetraxetan (2-[3-(1-Carboxy-5-{3-naphthalen-2-yl-2 -[(4-{[2-(4,7,10-tris- carboxymethyl-1,4,7,10-tetraaza-cyclododec-1-yl)-acetylamino]-methyl}-cyclohexanecarbonyl)-amino]-propionylamino}-pentyl)-ureido]-pentanedioic acid, further referenced as PSMA-617 (C_49_H_71_N_9_O_16_; MW = 1042.14 g/mol) was purchased from Advanced Biochemical Compounds (ABx, Radeberg, Germany) in a 1.0 mg batch with 99.0% radiolabeling precursor purity (colorless, solid powder). A 1850 MBq-loaded, tin-dioxide-based ^68^Ge/^68^Ga-generator was used for supply of radioactivity (iThemba LABS, Somerset West, South Africa). The animals’ blood samples were drawn and stored in BD Vacutainer, Fisher Scientific Inc. (Schwerte, Germany).

### 4.2. Preparation and Radioanalysis of [^68^Ga]Ga-PSMA-617 and [^68^Ga]Ga-PSMA-617-ME

The principle of the radiosynthesis for [^68^Ga]Ga^-^PSMA-617 and formulation into the water-in-oil-in-water (*w/o/w*) microemulsion was adopted from a previously described method [11]. Briefly, the generator-derived ^68^Ga-radioactivity was eluted by performing manual eluate fractionation as described by Breeman et al., 2005 [23] and measured in a dose calibrator (CRC15, Capintec Inc, Pittsburgh, PA, USA) using 0.6 M hydrochloric acid Sigma Aldrich (St Louis, MO, USA). All radioactive measurements were corrected for decay to the time of injection. A C18-SepPak (Microsep, Sandton, South Africa) cartridge-purified [^68^Ga]Ga-PSMA-617 solution was obtained. An aqueous solution of sodium oleate 0.1% *w/v* combined with PVA 2% (*w/v*) was prepared in a volume ratio of 1:1 and mixed by continuous stirring. Subsequently, Tween 80, d-α-tocopherol, and [^68^Ga]Ga-PSMA-617 were gradually added ultimately producing a clear [^68^Ga]Ga-PSMA-617-ME. Equally, a microemulsion mixture without [^68^Ga]Ga-PSMA-617 (replaced by saline) was produced to achieve a “blank microemulsion” (ME blank) as the reference. The quality control of [^68^Ga]Ga-PSMA-617 included optical inspection, HPLC, and instant-thin layer chromatography (ITLC) radioanalysis according to previously published methods [24].

#### Characterization of ME and ^68^Zn-PSMA-617-ME

Size distribution, droplet size measured by dynamic laser scattering (DLS), and zeta potential measured via Laser Doppler velocimetry were all performed using a Malvern Zetasizer Nano ZS (Malvern Instruments, Worcestershire, United Kingdom) using protocols from previously reported methods [11]. The pH and conductivity values of each microemulsion were measured at ambient temperature using a PC 8, Accsen pH and conductivity meter (Lasec, Midrand, South Africa). Decayed samples of the [^68^Ga]Ga-PSMA-617-ME (further referred to as ^68^Zn-PSMA-617-ME for clarity) were characterized and further evaluated for physical stability including visualization of clarity and phase separation.

### 4.3. Animal Study Design

**Figure 5:**
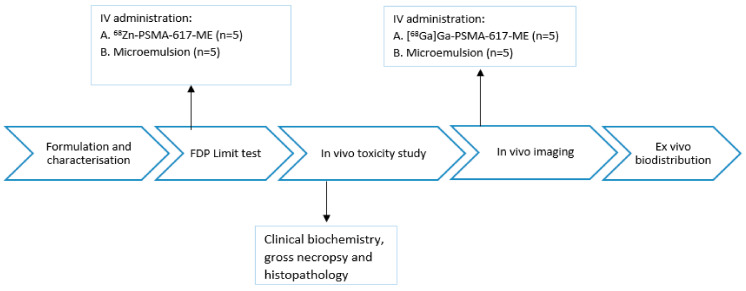
Study design assessing acute in vivo toxicity and tolerability of intravenously administered ^68^Zn-PSMA-617 and in vivo imaging of [^68^Ga]Ga-PSMA-617-ME.

The general study design is outlined in Figure 5. First, microemulsion and ^68^Zn-PSMA-617-ME were utilized for evaluation as described in the OECD guidelines to take into account the intravenous route of administration [25] along with endpoint analysis of selected parameters concerning food intake, clinical biochemistry gross necropsy, and histopathology. Subsequently, nuclear imaging and *post mortem* biodistribution with the radioactive equivalent, [^68^Ga]Ga-PSMA-617-ME, was performed to ascertain tolerability of the intravenous route of administration and yield information about the differences in acute pharmacokinetic behavior between [^68^Ga]Ga-PSMA-617-ME and [^68^Ga]Ga-PSMA-617 by way of dynamic and static microPET/CT imaging.

#### 4.3.1. Animal Studies

The North-West University Animal Care, Health and Safety Research Ethics Committee (NWU-AnimCare REC: NWU-00183-18-A5/-00333-15-A5) approved all animal related investigations prior to the study. Animals (BALB/c mice, male) were housed at a certified vivarium at North West University (Potchefstroom, South Africa) which forms part of the Department of Science and Technology/ Preclinical Drug Development Platform—a member of the Association for Assessment and Accreditation of Laboratory Animal Care (AAALAC) in South Africa. All mice were bred and obtained under specific pathogen-free conditions at the age of 5–6 weeks. Study animals were kept in an individual ventilated cage rack system (IVC Techniplast, Buguggiate, Italy) and were grouped based on breeding batch and behavior; the temperature in the housing facility was maintained at 21 ± 2 °C and a relative humidity of 55 ± 10% at a laboratory light/dark cycle of 12 h and room ventilation of 20 air changes per hour (under positive pressure) throughout the study. Animals were provided water *ad libitum* and fed standard rodent maintenance chow, and housed on bedding derived from dust free and non-toxic exfoliated corn cob chips.

#### 4.3.2. Acute In Vivo Toxicity and Tolerability of IV Administration of ^68^Zn-PSMA-617-ME

The study design was adapted from the OECD 420—fixed dose procedure (FDP) [25]. Ten animals were required for this study receiving a health check prior to the test. Upon enrolment into the study the mice were randomized into two treatment groups (*n* = 5 per group; variance in body weight of 10% acceptable). The animal handling, dosages administered, evaluation time frame, and key observations performed are consistent with previous reported literature [25] on oral or intravenous drug administration. Genotoxicity studies were deemed unnecessary based on the recommending guidelines [26]. Adaptations were made according to a particular consensus on radiopharmaceutical administration [26].

The *FDP Limit Test* was performed as recommended—first, using one animal for each compound and administering the highest dose feasible in a bolus volume of 150 µL (maximum injectable volume for intravenous administration in mice) [27], followed by dosing the other four animals/group. Upon injection, animals were kept on a warming pad and restrained during injection. The ME and ^68^Zn-PSMA-617-ME (Table 4) were administered intravenously via one of the lateral tail veins. The tolerability of the administration condition was observed by way of monitoring any immediate adverse reactions, vital signs or signs of distress during injection and after dosing at least once during the first 30 min, periodically during the first 24 h (at least every 4 h) and daily thereafter for 14 days.

*Body Weight and Food Consumption* was monitored daily, including animal health observations for the duration of the study. The general health check and monitoring at any given time point included the recording and reporting of the general physical condition (skin coat, eyes and mucous membranes), signs of respiratory malfunction, appearance of excretion (presence/absence of urinary symptoms and/or diarrhea), and changes in behavior (aggression/lethargy).

*Termination, Gross Necropsy, and Histopathology:* Animals were sacrificed by way of cervical dislocation on day 14 post injection. A gross necropsy was performed on each animal to determine any changes in organ/ tissue or size. Organs of each animal were isolated during the gross necropsy in a manner that ensured organ integrity. The isolated organs (heart, lungs, liver, spleen, kidneys, brain, stomach, small and large intestines) were stored in 10% formalin. Histopathology was performed as previously reported [28].

*Clinical biochemistry:* Ancillary blood testing for any long term effects of the treatment (at day 14 post injection) was performed; full blood samples were collected in SST II Advance tubes (Becton Dickenson, Franklin Lakes, NJ, USA) and analyzed directly after euthanasia. The blood provided per animal allowed for selected measurements; the levels of: (I) sodium and potassium (electrolytes), (II) urea and creatinine (kidney function), (III) selected albumin, bilirubin (serum protein), (IV) alkaline phosphatase (ALP), ALT, AST and amylase (hepatic/ pancreatic enzymes) and (V) total cholesterol, high density lipoproteins (HDL), low density lipoproteins (LDL), and triglycerides (blood lipids).

### 4.4. MicroPET/CT Imaging

MicroPET/CT imaging was performed using previously described animal handling procedures, type and modus of anesthesia, microPET/CT imaging equipment, image acquisition procedures, scan reconstruction protocols, and image analysis methods [11]. Ten male BALB/c mice (age: 6 weeks, body weight range: 25–30 g) underwent either [^68^Ga]Ga-PSMA-617-microPET/CT (*n* = 5) or [^68^Ga]Ga-PSMA-617-ME microPET/CT (*n* = 5). Prior to injection animals were anaesthetized in an animal chamber using a PrepaCell^TM^ unit (Mediso Medical Systems, Budapest, Hungary), to warrant adequate animal positioning, anesthesia control and animal monitoring (vital signs). Subsequently the animal chamber was transferred to the microPET/CT equipment and connected to the apparatus’ animal chamber holder which took over the monitoring. Once vital signs were stable, a topogram was utilized to adjust the animal for a microCT scan which was subsequently performed for the whole body. Without any further animal preparations either [^68^Ga]Ga-PSMA-617 or [^68^Ga]Ga-PSMA-617-ME were intravenously injected (0.15–0.20 mL per animal). Animals underwent immediate dynamic PET imaging for a duration of 40 min, then were allowed to recover before a second image acquisition was performed (static microPET scan from 90–120 min post injection).

### 4.5. Post Mortem Biodistribution

Following completion of the image acquisition each anesthetized animal was immediately euthanized by way of cervical dislocation. Animal dissection was performed immediately with relevant tissue/organs (blood, heart, lungs, liver, spleen, kidneys, intestines, stomach, urine, tissue, bone, and brain) being harvested, weighed, and further measured in an automated γ-counter (Hidex Gamma Counter AMG, Turku, Finland). The data (counts per minute) was corrected for decay and background and translated to radioactivity (kBq/sample). Results were reported as percent injected dose per gram (% ID/g) to compare all organs and tissues.

### 4.6. Statistical Analysis

If not otherwise referred to in the text, tests were performed in triplicates. Representative data and images may be shown for a larger population. Quantitative analysis was either reported as the range or expressed as mean ± standard deviation (SD) of mean or standard error of mean. Statistical analysis was carried out using the SAS software edition 9.4 (SAS Institute, Cary, NC, USA). A one-sided Mann–Whitney U-test was used to compare the two groups in terms of organ data. Where applicable, values were statistically validated using a Student’s *t* tests. *P* values <0.05 were considered statistically significant. A one-sided Mann-Whitney U-test (also called the Wilcoxon two-sample test) using the SAS statistical software (version 9.4) was used to compare the two treatment groups (i.e., ME vs. ^68^Zn-PSMA-617-ME). This test is more appropriate to use than the Student’s *t*-test to test for statistically significant differences in small sample populations.

## 5. Conclusions

An optimized procedure for the [^68^Ga]Ga-PSMA-617 formulation into a ME is presented featuring high radiochemical yields and optimal pH for intravenous administration. Adequate stability and integrity of the formulation matched with the activity dose and injectable volume for further investigation in mice. The safety profile for the intravenous administration assured demonstration of no adverse toxic effects neither for the ME (blank) nor the [^68^Ga]Ga-PSMA-617-ME during a 14-day observation period. No adverse signs of distress, indications of intoxication, or significantly altered blood biochemistry occurred. Subsequently, [^68^Ga]Ga-PSMA-617-ME doses were tolerated by male BALB/c mice directly following the injection, which is of unprecedented nature. [^68^Ga]Ga-PSMA-617-ME featured an unchanged clearance from the blood pool and slightly delayed peak activity in kidneys compared to [^68^Ga]Ga-PSMA-617. These findings support that MEs can be considered safe delivery systems for intravenous administration of particular drug-like compounds [29].

However, it would be too presumptuous to conclude on the ME achieving the desired outcome in this study; a clear distinction of the biodistribution profile of [^68^Ga]Ga-PSMA-617 and [^68^Ga]Ga-PSMA-617-ME is debatable. While the in vivo data are preliminary, it is already observable that the postulated effects are not being achieved and that this approach seems not particularly valuable to impart benefits on PSMA-617-based radiotherapy. However, a similar concept of a delivery system to improve drug efficacy and clinical outcomes could still be investigated on radiometal-based nanoparticles using isotopes that are not limited by a short half-life.

## Figures and Tables

**Table 1:** Physicochemical characteristics of formulations used in animal studies.

Sample *	F	Size (nm)	PDI	ZP (mV)	pH	Conductivity (µS/cm)
**^68^Zn-PSMA-617**	1	25.13 ± 0.02	0.35 ± 0.01	−26.25 ± 0.53	6.50	13.01
	2	22.25 ± 0.31	0.34 ± 0.00	−22.10 ± 3.55	6.89	12.20
	3	25.79 ± 0.13	0.32 ± 0.00	−27.22 ± 0.16	6.90	13.19
**^68^Zn-PSMA-617-ME**	4	30.48 ± 5.06	0.34 ± 0.06	−19.25 ± 0.53	7.50	14.21
	5	58.43 ± 5.25	0.25 ± 0.01	−26.34 ± 0.29	7.00	15.01
	6	52.87 ± 0.38	0.44 ± 0.00	−27.96 ± 0.47	7.40	14.87

Results are presented as mean (± standard deviation (SD); *n* = 5). F = formulation; PDI = polydispersity index; ZP = zeta potential; * representative preparation for intravenous administration to animal, tested after radioactive decay.

**Table 2:** Comparison of organ weights (g) following gross necropsy of BALB/c mice.

Sample	ME (blank)	^68^Zn-PSMA-617-ME
Heart	0.13 ± 0.03	0.14 ± 0.01
Lungs	0.17 ± 0.03	0.18 ± 0.02
Liver	0.92 ± 0.22	1.01 ± 0.10
Spleen	0.05 ± 0.01	0.05 ± 0.01
Stomach and SI	1.28 ± 0.29	1.21 ± 0.16
Large intestine	0.22 ± 0.04	0.27 ± 0.08
Kidneys2	0.30 ± 0.05	0.33 ± 0.03
Brain	0.35 ± 0.03	0.35 ± 0.05

Results are presented as mean (±SD; *n* = 5); SI = small intestines.

**Table 3:** Summary of clinical biochemistry (day 14 after compound injection).

	Normal *	ME	^68^Zn-PSMA-617-ME	Control
**Serum Proteins**				49
Total protein (g/L)	4–70	49	48 ± 1	
Albumin (g/L)	21–30	**31**	27	27
Bilirubin (umol/L)	0–1	-	**2.0** ± 0.02	-
Phosphatase (IU/L)	-	**359**	210 ± 60	157
**Hepatic Function**				34
ALT (IU/L)	10–35	27 ± 2	**38** ± 5	
AST (IU/L)	54–298	-	135	110
**Pancreatic Function**				-
Amylase (IU/L)	1496–3200	**1494**	-	
**Kidney Function**				**13**
Urea (mmol/L)	4–11	8 ± 1	11 ± 1	
Creatinine (umol/L)	40–70	**19**	31 ± 10	50
**Electrolytes**				
Na^+^ (mmol/L)	140–160	149 ± 2	**138** ± 7	145
K^+^ (mmol/L)	5–8	7.4	6.4	-
**Serum Lipids**				2.7
Cholesterol (mmol/L)	5–7	-	**2.0** ± 0.1	
HDL (mmol/L)	-	1.6 ± 0.1	1.6 ± 0.1	2.10
LDL (mmol/L)	-	-	0.3	-
Triglycerides (mmol/L)	5–10	**1.0**	**1.2** ± 0.3	1.7

Mean (±SD, whenever *n* = 3) (*): normal range was described previously [12,13]; (-): non-comparative analysis was performed due to insufficient blood sample volume; Bold: indicator for an out-of-range value.

**Table 4:** Specifications of the microemulsion formulations employed in the toxicity study.

Test Formulation:	ME	Composition of Formulation (*v/v*)
**Size:**	72.23 nm ± 0.18	1. Lauric acid-PEG 4000 (0.17%)
**PDI:**	0.26 ± 0.005	2. Ethanol (8.28%)
**Zeta Potential:**	−2.87 mV ± 0.53	3. Polyvinyl alcohol (41.39%)
**pH:**	6.89	4. Sodium oleate (41.39%)
**Appearance:**	Golden-translucent liquid	5. d-α-tocopherol (0.17%)
		6. Tween 80 (0.33%) 7. Saline (8.28%)
**Test Formulation:**	[^68^Ga]Ga-PSMA-617-ME	**Composition of Formulation (*v/v*)**
**Size:**	27.61 nm ± 1.11 nm	1. Lauric acid-PEG 4000 (0.17%)
**PDI:**	0.53 ± 0.006	2. Ethanol (8.28%)
**Zeta Potential:**	−2.31 ± 1.49 mV	3. Polyvinyl alcohol (41.39 %)
**pH:**	7.00	4. Sodium oleate (41.39%)
**Appearance:**	Golden-translucent liquid	5. d-α-tocopherol (0.17%)
		6. Tween 80 (0.33%)
		7. [^68^Ga] Ga-PSMA-617 (8.28%)

## Data Availability

The data presented in this study are available on request from the corresponding author. The data are not publicly available due to certain file restrictions.

## Sample Availability

Samples of the compounds are available from the authors.
